# Identification of Prognostic Gene Biomarkers in Non-Small Cell Lung Cancer Progression by Integrated Bioinformatics Analysis

**DOI:** 10.3390/biology10111200

**Published:** 2021-11-18

**Authors:** Panagiotis Giannos, Konstantinos S. Kechagias, Annamaria Gal

**Affiliations:** 1 School of Applied Sciences, University of Brighton, Lewes Road, Brighton BN2 4GJ, UK; 2 Department of Life Sciences, Faculty of Natural Sciences, Imperial College London, South Kensington, London SW7 2AZ, UK; 3 Department of Metabolism, Digestion and Reproduction, Faculty of Medicine, Imperial College London, London W12 0NN, UK; konstantinos.kechagias18@imperial.ac.uk

**Keywords:** non-small cell lung cancer, epithelial-mesenchymal transition, TGF-β, integrated bioinformatics analysis, gene biomarkers

## Abstract

**Simple Summary:**

Non-small cell lung cancer (NSCLC) is a major contributor to cancer related deaths worldwide. The progression of NSCLC is linked to epithelial-mesenchymal transition (EMT), a biologic process that enables tumor cells to acquire an invasive phenotype and resistance to therapies. Discovery of novel biomarkers in NSCLC progression is essential for improved prognosis and pharmacological interventions. We performed an integrated bioinformatics analysis on available gene expression datasets of transforming growth factor β (TGF-β) induced EMT in NSCLC cell lines aiming to establish new prognostic biomarkers in the disease. The retrieved candidate genes were involved in protein modifications, regulation of cell death and cell adhesions, oxidation-reduction reactions of aerobic respiration and mitochondrial translation. Out of these genes, we identified ten prognostic gene biomarkers, mostly involved in protein modifications, whose expressions correlated with patient survival in NSCLC. This ten-gene prognostic signature will be useful to improve risk prediction and guide treatment strategies in NSCLC. Deciphering the exact functions of the biomarker genes previously not linked with NSCLC will also lead to a better understanding of the pathomechanism of NSCLC progression, revealing novel therapeutic targets in the disease.

**Abstract:**

The progression of non-small cell lung cancer (NSCLC) is linked to epithelial-mesenchymal transition (EMT), a biologic process that enables tumor cells to acquire a migratory phenotype and resistance to chemo- and immunotherapies. Discovery of novel biomarkers in NSCLC progression is essential for improved prognosis and pharmacological interventions. In the current study, we performed an integrated bioinformatics analysis on gene expression datasets of TGF-β-induced EMT in NSCLC cells to identify novel gene biomarkers and elucidate their regulation in NSCLC progression. The gene expression datasets were extracted from the NCBI Gene Expression Omnibus repository, and differentially expressed genes (DEGs) between TGF-β-treated and untreated NSCLC cells were retrieved. A protein-protein interaction network was constructed and hub genes were identified. Functional and pathway enrichment analyses were conducted on module DEGs, and a correlation between the expression levels of module genes and survival of NSCLC patients was evaluated. Prediction of interactions of the biomarker genes with transcription factors and miRNAs was also carried out. We described four protein clusters in which DEGs were associated with ubiquitination (Module 1), regulation of cell death and cell adhesions (Module 2), oxidation-reduction reactions of aerobic respiration (Module 3) and mitochondrial translation (Module 4). From the module genes, we identified ten prognostic gene biomarkers in NSCLC. Low expression levels of *KCTD6*, *KBTBD7*, *LMO7*, *SPSB2*, *RNF19A*, *FOXA2*, *DHTKD1*, *CDH1* and *PDHB* and high expression level of *KLHL25* were associated with reduced overall survival of NSCLC patients. Most of these biomarker genes were involved in protein ubiquitination. The regulatory network of the gene biomarkers revealed their interaction with tumor suppressor miRNAs and transcription factors involved in the mechanisms of cancer progression. This ten-gene prognostic signature can be useful to improve risk prediction and therapeutic strategies in NSCLC. Our analysis also highlights the importance of deregulation of ubiquitination in EMT-associated NSCLC progression.

## 1. Introduction

Lung cancer is the leading cause of cancer-related deaths across the globe. Its high mortality rate is due to advanced stages of the disease at the time of diagnosis [1,2]. Non-small cell lung cancer (NSCLC) accounts for approximately 85% of all lung cancer cases and includes the histological subtypes of adenocarcinoma (LUAD), squamous cell carcinoma (LUSC) and large cell carcinoma [3]. Long-term survival of patients diagnosed with either subtype is poor due to local recurrence of the tumor and the development of metastatic lesions after complete resection [4,5].

In NSCLC progression, epithelial-mesenchymal transition (EMT) has been described as a key process, endowing cancer cells with enhanced motility, invasiveness, resistance to apoptosis and acquisition of stem cell-like properties which further enhance tumor survival [5,6,7]. Accumulating evidence has highlighted an association between EMT and resistance to anti-cancer therapies [8,9]. Sustained by hypoxia and cellular stress, EMT is induced by a plethora of signaling molecules, including epidermal growth factor (EGF), hepatocyte growth factor (HGF) and fibroblast growth factor (FGF) [10,11,12].

Transforming growth factor beta (TGF-β) is considered one of the most potent inducers of EMT, both in vitro and in vivo, exerting a critical tumor-promoting function in advanced stages of NSCLC [13,14]. Secreted by both the cancer cells and the cellular components of the tumor microenvironment, TGF-β also acts as a regulator of multiple biological processes essential in NSCLC progression, including angiogenesis, immunoevasion and immunosuppression [15,16]. While the effect of TGF-β is context dependent, acting as a tumor suppressor at the early stages, its expression level correlates with tumor progression and metastasis [17,18]. Genetic variations in the TGF-β1 signaling pathway can improve prediction of overall survival of patients with NSCLC [19]. Moreover, a monoclonal antibody against TGF-β1, 2 and 3 (fresolimumab), vaccines targeting TGF-β signaling (Lucanix^TM^, FANG^TM^), and a small molecule inhibitor of the TGF-β receptor I (galunisertib) are in clinical trials for the treatment of NSCLC [20].

Attributed to the biological complexity and poor prognosis of the disease, not all patients who are positive for acknowledged biomarkers of NSCLC (e.g., mutated EGFR, ALK, or ROS1) benefit from existing molecular therapies [21]. Therefore, integrated bioinformatics analysis of available gene expression datasets on NSCLC meets the current clinical needs for novel prognostic biomarkers that can inform therapeutic decision-making.

In a previous study, a 16-gene EMT signature was found inversely associated with T-cell infiltration in NSCLC [22]. A TGF-β-induced EMT gene signature was also reported to predict significantly worse metastasis-free survival of NSCLC patients [23]. High expression of EGFR- and EMT-related proteins was shown in the peripheral leading edge of NSCLC samples and found associated with poor prognosis [24]. In our study, we conducted an integrated bioinformatics analysis on available microarray datasets of TGF-β-induced EMT in NSCLC cells, which increases statistical power and robustness of the results retrieved. Our aim was to identify potential gene biomarkers strongly correlated with the progression of NSCLC, informing overall survival, survival until first progression and pots (first) progression survival of patients.

## 2. Methods

### 2.1. Data Sources and Search Strategy

The National Center for Biotechnology Information (NCBI) Gene Expression Omnibus (GEO) database was searched using the keyword “epithelial-mesenchymal transition”. Two authors (P.G. and K.S.K.) searched the database independently, and no language restrictions were included for article retrieval. Studies were selected according to organism type (Homo sapiens), gene expression profiling (microarray), cancer type (NSCLC) and in vitro treatment modality of EMT initiation (TGF-β supplementation).

### 2.2. Identification of Differentially Expressed Genes

Identification of differentially expressed genes (DEGs) from the pre-normalized pooled microarray profiles of NSCLC cells with TGF-β treatment vs. untreated NSCLC cells was performed using ImaGEO [25]. Integration of DEGs was conducted via the random effect model which combines effect sizes across all datasets into a meta-effect size. Whilst incorporating cross-study heterogeneity, DEGs with the strongest average effect across all studies were identified. Genes with *p* < 0.05 corrected by the Benjamini-Hochberg (BH) false discovery rate (FDR) were considered significant. DEGs were regarded upregulated based on z > 1.96, while considered downregulated with z < −1.96 (both corresponding to a 5% significance level). Across the gene expression datasets, the homogeneity and heterogeneity magnitudes of each significant DEG were quantified using Cochran’s Q test and Tau squared (*τ*^2^). DEGs based on P_Cochran’s Q_ > 0.05 and *τ*^2^ = 0 were considered highly homogeneous.

### 2.3. GO Functional and KEGG Pathway Enrichment

Gene Ontology (GO) and Kyoto Encyclopedia of Genes and Genomes (KEGG) pathway enrichment analyses of total and module DEGs were conducted using the ToppGene Suite [26]. Functional enrichment was categorized into three groups of GO terms: biological process (BP), molecular function (MF) and cellular component (CC). Enriched GO terms and KEGG pathways with a *p* < 0.05, corrected by BH FDR, were considered significant.

### 2.4. Protein-Protein Interaction Network Construction and Module Analysis

Network construction of proteins encoded by the total DEGs with a probabilistic confidence score > 0.4 was attained using the Search Tool for the Retrieval of Interacting Genes and Proteins (STRING) database followed by visualization with Cytoscape [27,28]. Protein nodes lacking a connection in the network were excluded.

Protein-protein interaction (PPI) network-based clustered modules were retrieved using the Molecular Complex Detection (MCODE) tool [29]. Modules with MCODE score ≥ 5.5 and nodes ≥ 5 were considered significant. Protein nodes with a higher number of incident edges were regarded hubs according to a degree centrality index ≥ 11.0 using the CentiScaPe plugin [30].

### 2.5. Construction of Gene Regulatory Network

Prediction of transcription factor and miRNA-gene interactions was obtained using MSigDB and TargetScan [26,31,32]. A gene regulatory network (GRN) of the biomarkers, depicting their interactions with transcription factors and miRNAs, was constructed using Cytoscape [27]. Enriched transcription factors and miRNAs with a *p* < 0.01, corrected by BH FDR, were considered significant.

### 2.6. Survival Analysis

Correlation between the expression levels of module genes and survival of patients with NSCLC was evaluated using the Kaplan-Meier-plotter [33]. Gene expression profiles of NSCLC tumor samples of 3251 patients from GEO, The Cancer Genome Atlas (TCGA) and caArray databases were sourced to obtain and verify the prognostic values of the module genes. Patients were divided into groups with high or low gene expression based on auto-selected best cut-off whereby each possible cut-off between the lower and upper quartiles was examined and the most robust threshold was selected. Module DEGs associated with reduced survival until first progression, post (first) progression survival and overall survival upon a log-rank test *p* < 0.001 and corrected *p* < 0.05 by BH FDR for the patient cut-off selection method were considered significant and presented as potential gene biomarkers in NSCLC progression.

## 3. Results

### 3.1. Overview of the Datasets Included in the Analysis

**Figure 1:**
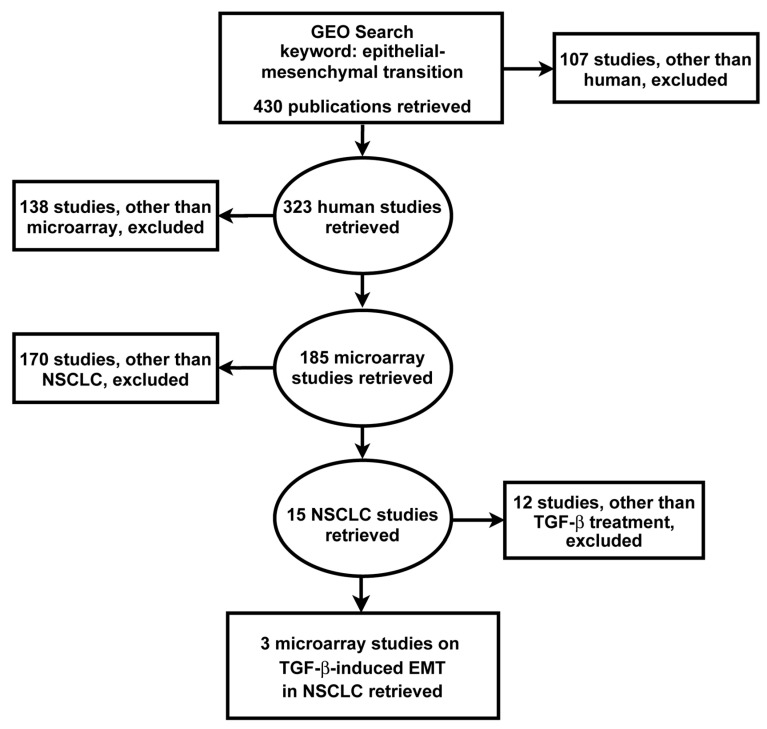
Search strategy for the identification, screening and selection of eligible gene expression studies. Searching the GEO yielded 430 studies which were screened by applying the indicated exclusion criteria. Three microarray datasets met the search criteria for subsequent integrated bioinformatics analysis of TGF-β-induced EMT in NSCLC cells.

From the GEO database, 430 EMT studies were retrieved, of which 185 microarray studies were obtained having excluded other types of gene expression datasets. Further exclusion based on cancer types resulted in 15 NSCLC studies. Exclusion based on treatment modality yielded three independent gene expression studies of TGF-β-induced EMT in NSCLC, incorporating the microarray datasets GSE17708, GSE42373 and GSE49644 (Figure 1) [34,35,36,37].

All three gene expression studies used the A549 cell line, and one also included the HCC827 and NCI-H358 cell lines, all LUAD, the most common subtype of NSCLC, and responsive to TGF-β (Table 1). In one study, TGF-β treatment was preceded by TNF-α treatment to initiate EMT. The duration of TGF-β treatment varied across the studies, ranging from 0.5 h to 3 weeks (Table 1).

### 3.2. Identification of Functions and Pathways in NSCLC Progression

The pre-normalized microarray datasets were subjected to a significance analysis using the random effect model to reveal genes with significantly altered expressions between the test (TGF-β-treated) and the control (untreated) NSCLC cell samples. A total of 725 DEGs were obtained, among which 566 were highly homogenous. Of these, 215 genes were upregulated (z > 1.96) and 351 genes were downregulated (z < −1.96) (Figure S1, Tables S1 and S2).

**Figure 2:**
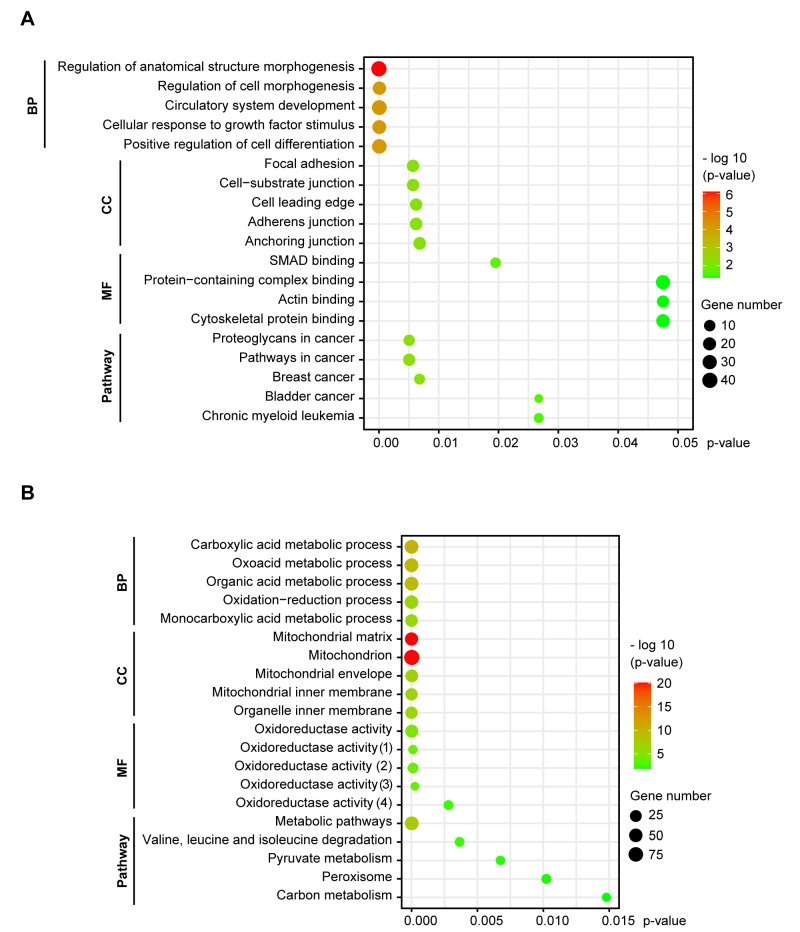
GO functional and KEGG pathway enrichment of DEGs in NSCLC cells undergoing EMT. Functional enrichment of DEGs was categorized into three groups of GO terms: biological process (BP), cellular component (CC) and molecular function (MF). The significance threshold was set to *p* < 0.05, corrected by BH FDR. (**A**) The upregulated genes were involved in morphogenesis, cell adhesions, SMAD and cytoskeleton binding, and associated with cancer pathways. (**B**) The downregulated genes were linked with metabolic processes, the mitochondrion, oxidoreductase activity and metabolic pathways. (1) Acting on a sulfur group of donors, NAD(P) as acceptor; (2) Acting on NAD(P)H; (3) Acting on the aldehyde or oxo group of donors, disulfide as acceptor; (4) Acting on a sulfur group of donors.

The upregulated genes were associated with regulation of morphogenesis, cellular response and differentiation (BP enrichment), focal adhesion and cell junctions (CC enrichment) and SMAD and cytoskeletal protein binding (MF enrichment). KEGG pathway mapping revealed an association of the upregulated genes with proteoglycans and pathways in cancer (Figure 2A). The downregulated genes were linked with metabolic and oxidation-reduction processes (BP), the mitochondrion (CC) and oxidoreductase activity (MF). Pathway enrichment connected the downregulated genes with metabolic pathways (Figure 2B). The upregulated genes point to the morphological changes NSCLC cells undergo to gain a mesenchymal phenotype, while the downregulated genes indicate that a complex metabolic rewiring is taking place in EMT, consistent with increasing evidence from recent studies [38].

### 3.3. Protein-Protein Interaction Network and Module Genes in NSCLC Progression

**Figure 3:**
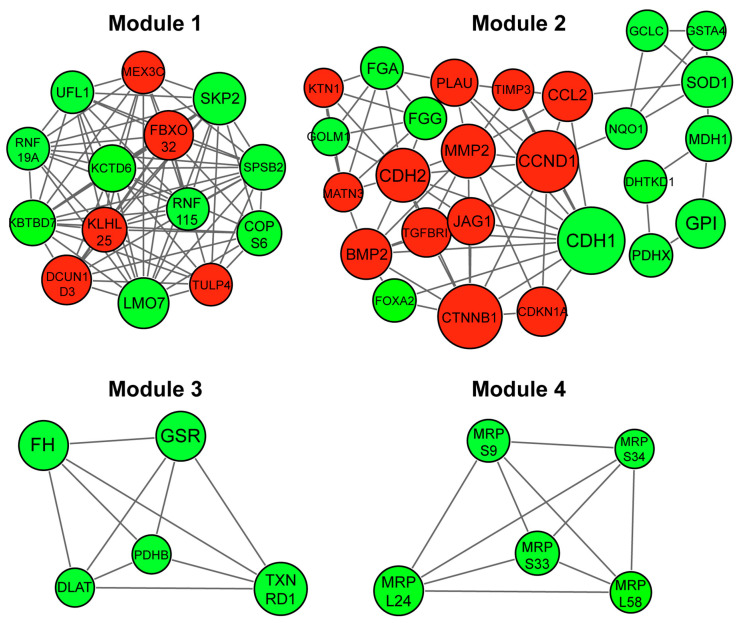
Module analysis of protein-protein interaction (PPI) network of DEGs in EMT of NSCLC cells. PPI network-based clustered modules were retrieved and clustering modules with a combined node interaction score of > 0.4 and MCODE score ≥ 5.5 and nodes ≥ 5 are displayed. Red indicates upregulated and green shows downregulated node genes. Larger nodes show hub genes with a centrality degree of ≥12.0. Module 1 (score = 12.154), Module 2 (score = 6.000), Module 3 (score = 5.000) and Module 4 (score = 5.000).

A network was constructed based on predicted interactions between proteins encoded by the DEGs. A total of 541 nodes and 1263 edges were obtained with a combined score > 0.4, including 170 upregulated and 286 downregulated genes (Figure S2). Among these, 60 hub gene candidates with a degree centrality index of ≥11.0 were identified. The complete PPI network was divided into highly dense clustering modules. Among the total 14 modules retrieved, four significant modules with MCODE score ≥ 5.5 and nodes ≥ 5 were identified and the fold changes (z-scores) of DEGs indicated (Figure 3, Table S3).

**Figure 4:**
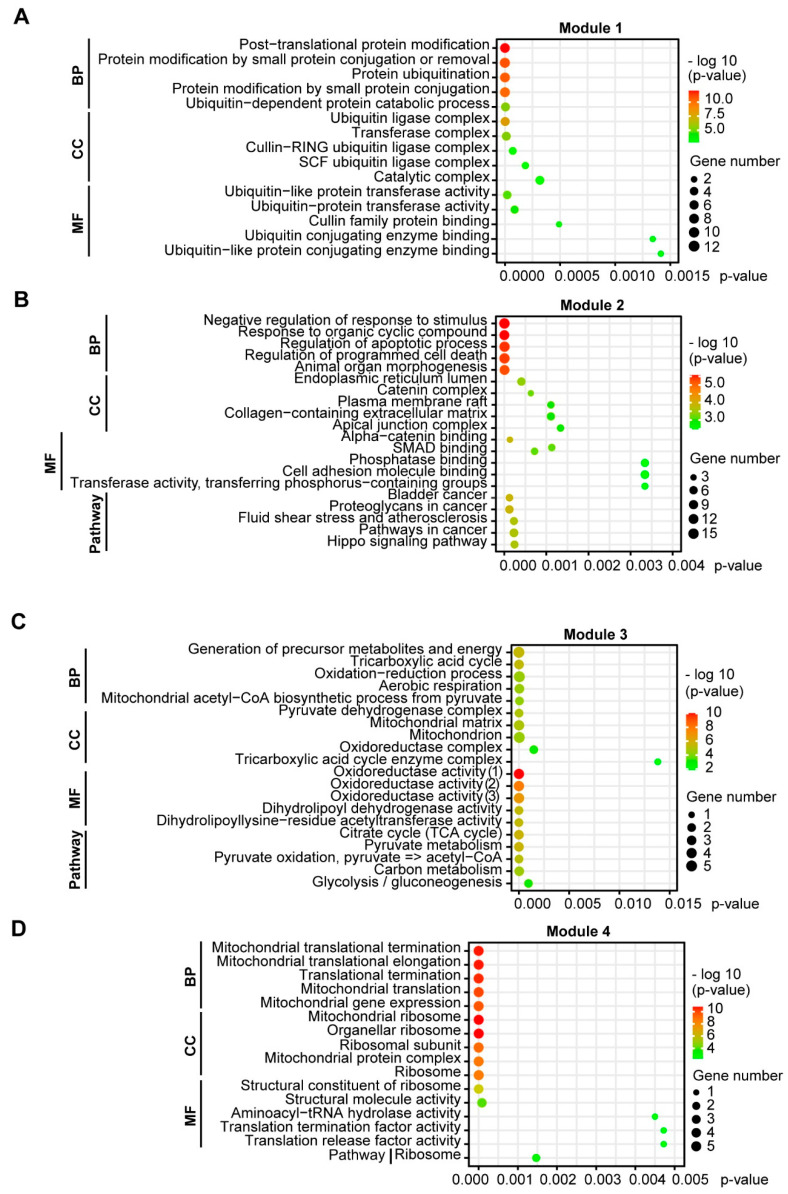
GO functional and KEGG pathway enrichment of clustering module DEGs in EMT of NSCLC cells. Functional enrichment of DEGs was categorized into three groups of GO terms: biological process (BP), cellular component (CC) and molecular function (MF). The significance threshold was set to *p* < 0.05, corrected by BH FDR. (**A**) Module 1 was involved in protein modification and ubiquitination. (**B**) Module 2 was linked with regulation of programmed cell death and morphogenesis, cell adhesions and pathways in cancer. (**C**) Module 3 was associated with aerobic respiration, the mitochondrial matrix, and pathways in glycolysis, pyruvate metabolism and the TCA cycle. (**D**) Module 4 related to mitochondrial translation and the mitochondrial ribosome.

Functional GO enrichment of the four modules revealed that Module 1 was involved in protein modification and ubiquitination via, e.g., the Cullin-RING (CLR) and Skp, Cullin, F-box (SCF) ubiquitin ligase complexes (Figure 4A). Module 2 related to regulation of programmed cell death, morphogenesis, cell adhesions, SMAD and phosphatase binding (Figure 4B). Module 3 was associated with generation of energy/aerobic respiration, the mitochondrial matrix and oxidoreductase activity (Figure 4C). Module 4 was connected with mitochondrial translation and the mitochondrial ribosome (Figure 4D). KEGG pathway enrichment of the modules revealed that Module 2 was associated with pathways in cancer, including the Hippo signaling pathway, while Module 3 was involved in glycolysis, pyruvate metabolism and the tricarboxylic acid (TCA) cycle (Figure 4B,C).

### 3.4. Prognostic Gene Biomarkers in NSCLC

**Figure 5:**
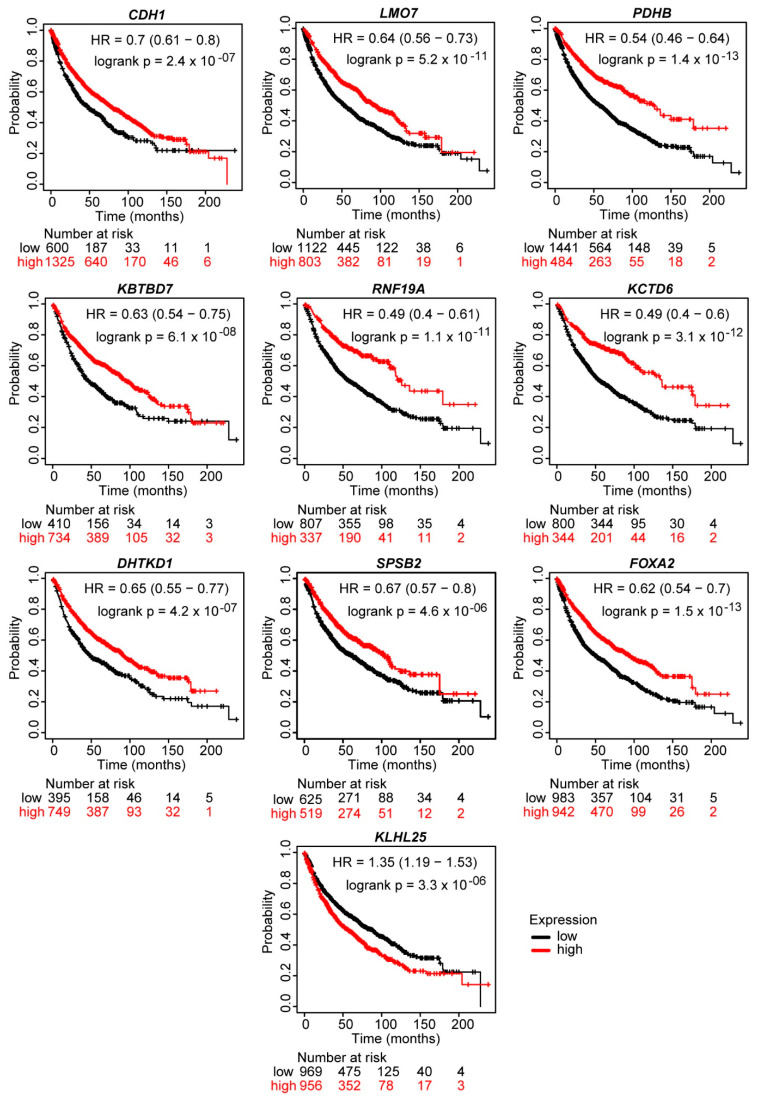
Prognostic value of genes associated with EMT in NSCLC. Kaplan-Meier association between the expression levels of module DEGs and overall survival of patients with NSCLC (*n* = 1144) is displayed. Significance thresholds for potential gene biomarkers include a log-rank test *p* < 0.001 and a corrected *p* < 0.05 by BH FDR for the patient cut-off selection method. Low expression levels of *CDH1*, *LMO7*, *PDHB*, *KBTBD7*, *RNF19A*, *KCTD6*, *DHTKD1*, *SPSB2* and *FOXA2* while high expression level of *KLHL25* were associated with reduced overall survival.

The prognostic value of potential biomarkers in the module gene sets was determined using gene expression profiles of NSCLC tumor samples from 3251 patients (Table S4). Low expression levels of nine genes, including forkhead box A2 (*FOXA2*), potassium channel tetramerization domain containing 6 (*KCTD6*), kelch repeat and BTB domain containing 7 (*KBTBD7*), dehydrogenase E1 and transketolase domain containing 1 (*DHTKD1*), LIM domain 7 (*LMO7*), pyruvate dehydrogenase E1 beta subunit (*PDHB*), splA/ryanodine receptor domain and SOCS box containing 2 (*SPSB2*), E-cadherin (*CDH1*) and ring finger protein 19A (*RNF19A*), and high expression level of the kelch-like family member 25 (*KLHL25*) were associated with significantly reduced overall survival (Table 2, Figure 5).

Low expression levels of *CDH1*, *LMO7*, *PDHB*, *RNF19A* and *FOXA2* and high expression level of *KLHL25* were associated with reduced survival until first progression (Figure S3). Low expression levels of *PDHB*, *KBTBD7*, *RNF19A*, *KCTD6* and *FOXA2* were linked to decreased post-progression survival (Figure S4).

Most of the gene biomarkers (*KLHL25*, *KCTD6*, *KBTBD7*, *LMO7*, *SPSB2* and *RNF19A*) were associated with ubiquitination (Table 2), suggesting an important role of the process in NSCLC progression.

### 3.5. Gene Regulatory Network of the Proposed Gene Biomarkers

**Figure 6:**
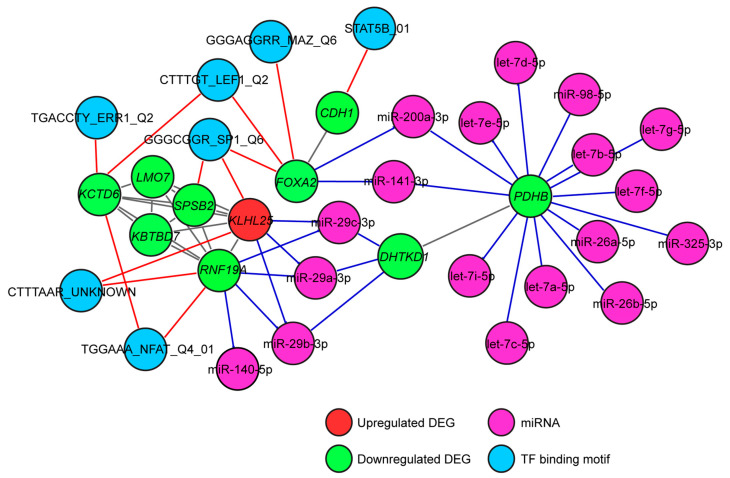
Gene regulatory network of the proposed gene biomarkers in NSCLC progression. A gene regulatory network for the biomarkers was constructed, and enriched transcription factors and miRNAs with a *p* < 0.01, corrected by BH FDR, were considered significant. Transcription factors associated with tumor progression and tumor suppressor miRNAs were found involved in the regulation of the gene biomarkers.

To shed light on the regulation of the gene biomarkers, an interaction network of the biomarker DEGs with transcription factors and miRNAs was constructed. Tumor suppressor miRNAs, including the let-7 and miR-26 family members [39,40,41] were found to be prominent in the regulation of *PDHB* (Figure 6). Two of the *PDHB*-interacting miRNAs, miR-200a-3p and miR-141-3p [42,43], were also involved in the regulation of *FOXA2*. *DHTKD1* was found interacting with the tumor suppressive miR-29 family members [44,45,46]. These miR-29 family members appeared to also regulate *KLHL25*, and one of them, miR-29b-3p, was interacting with *RNF19A* too (Figure 6).

Transcription factors associated with various mechanisms of lung cancer progression were also involved in the regulation of the biomarker genes [47,48,49,50]. SP1 was found regulating *FOXA2*, *SPSB2* and *KLHL25*. NFAT, ERR1, LEF1 and MAZ were linked with the downregulated gene biomarkers only, and STAT5B was the only transcription factor associated with the regulation of *CDH1* (Figure 6). No connection of the biomarker genes with EMT transcription factors (EMT-TFs) such as SNAIL, SLUG or TWIST was revealed in our analysis.

## 4. Discussion

Our integrated bioinformatics analysis on microarray datasets of TGF-β-induced EMT in NSCLC cells revealed four significant protein clusters within the 215 upregulated and 351 downregulated DEGs. These modules were associated with protein ubiquitination (Module 1), morphogenesis and cell adhesions (Module 2), oxidoreductase activity of aerobic respiration (Module 3) and mitochondrial translation (Module 4) confirming the significance of these processes in the regulation of EMT.

We found ten genes correlated with overall survival of patients with NSCLC, and only one of them (*CDH1*) was a canonical EMT-marker. Low expressions of nine gene biomarkers were associated with decreased survival. Most of them, including *KCTD6*, *KBTBD7*, *LMO7*, *SPSB2* and *RNF19A* were Module 1 genes, involved in protein modifications and ubiquitination [51,52,53,54,55].

In an earlier study, *KBTBD7* which regulates extracellular-cytoskeletal signal transduction, appeared to be protective in early-stage NSCLC as one of an 8-gene prognostic signature [55,56]. *LMO7* which regulates the actin cytoskeleton and adherens junctions, was described to be downregulated in malignant lung tissue, and *LMO7* deficiency was found associated with genetic predisposition to lung cancer [51,57].

The downregulation of *FOXA2*, *DHTKD1* and *CDH1*, involved in embryonic development, oxidation reactions and intercellular adhesions, respectively, were also linked to worse prognosis of NSCLC patients in our study.

*FOXA2* was reported to inhibit EMT and suppress metastasis in human lung cancer cell lines via repressing the SLUG promoter [58]. Loss of *FOXA2* expression was found frequent in lung cancer cell lines and NSCLC tumor samples [59].

So far, no correlation of *DHTKD1* (component of a mitochondrial 2-oxoglutarate-dehydrogenase-complex) expression with survival of NSCLC patients has been described. In breast carcinoma, differential DNA methylation between tumors and normal tissues was found correlated with the expression level of *DHTKD1* [60].

*CDH1*, a tumor suppressor gene encoding E-cadherin, plays a crucial role in maintaining intercellular junctions in the epithelium [61]. In an earlier study, downregulation of E-cadherin was found to promote *EGFR* transcription in NSCLC [62]. In NSCLC patients, the mRNA level of the Wilms tumor gene (*WT1*) was reported to be negatively correlated with that of *CDH1* and was associated with pathological stage, metastasis, and survival rate [63]. High E-cadherin and low vimentin expression was linked to better overall survival of NSCLC patients [64].

*PDHB*, the mitochondrial pyruvate dehydrogenase E1 component subunit beta, a Module 3 gene in our study, catalyzes the decarboxylation of pyruvate to acetyl-CoA, linking the glycolytic pathway to the TCA cycle [65]. PDBH was described as a biomarker within energy metabolism heterogeneity of ovarian cancer cells for the diagnosis and prognosis of ovarian cancer [66]. We found the downregulation of *PDHB* linked to worse prognosis in NSCLC.

In our integrated bioinformatics analysis, *KLHL25* was the only gene whose high expression was associated with a significantly reduced overall survival of NSCLC patients, thus representing an unfavorable prognostic marker in NSCLC progression.

*KLHL25*, a member of the Kelch-like (KLHL) gene family, a substrate-specific adaptor in the cullin-3 (Cul3)-dependent ubiquitin ligase complex, is required for eukaryotic translational control [67]. Mutations in four of the Kelch-family members have been linked to cancer [68]. However, the exact mechanism how *KLHL25* contributes to NSCLC progression warrants further investigations.

Although we found key members of the zinc-finger transcription factor family upregulated in TGF-β-induced EMT in NSCLC cells (*ZEB1*, z-score = 4.40 and adj. *p* = 1.09 × 10^−5^; *SNAI2*, z-score = 3.07 and adj. *p* = 4.83 × 10^−2^), binding motifs of EMT-TFs were not significantly enriched in the gene regulatory network, suggesting no direct interaction of EMT-TFs with *CDH1* or the other proposed gene biomarkers in NSCLC progression. Our findings are consistent with recent evidence showing that EMT-TFs also act via indirect mechanisms to downregulate E-cadherin and induce EMT and NSCLC progression [69,70].

Overall, to clarify the role of our biomarker genes and their regulation in NSCLC progression, further validation and functional studies are required. The limitation of our study is the lack of such biological experiments.

Of the gene biomarkers we propose in NSCLC progression, *KBTBD7* has already been described as a favorable prognostic indicator in NSCLC [55,56]. An association between the deficiency of *LMO7* and genetic predisposition to lung cancer has also been reported [57]. Moreover, *CDH1* and *FOXA2* are linked to each other in carcinoma progression, evidenced by the loss of silencing *FOXA2* leading to E-cadherin downregulation, EMT and metastasis [71,72]. However, most of the gene biomarkers we put forward have not been linked to NSCLC prognosis so far.

Our results also reveals that deregulation of protein ubiquitination has an important function in EMT-associated NSCLC progression. Furthermore, deciphering the function of *KLHL25* in the pathomechanism of NSCLC may lead to new therapeutic targets.

## 5. Conclusions

Genes regulated during EMT of NSCLC cells are closely linked to NSCLC progression. The ten prognostic gene biomarkers we obtained via an integrated bioinformatics analysis of EMT-associated gene expression data can provide improved risk prediction and lead to new therapeutic targets in NSCLC.

## Figures and Tables

**Table 1:** Description of the microarray datasets selected for the analysis.

**GEO Dataset ID**		GSE17708	GSE42373	GSE49644
**References**		Sartor et al., 2010 [34]	Cieślik et al., 2013 [35]; Wamsley et al., 2015 [36]	Sun et al., 2014 [37]
**Cell lines**		A549	A549	A549, HCC827, NCI-H358
**Histopathology classification**		LUAD (NSCLC)		
**EMT inducer**		TGF-β1	TGF-β1 *	TGF-β **
**Treatment**	**Concentration**	5 ng/mL	2 ng/mL	2 ng/mL
	**Duration**	0.5–72 h	48 h	3 weeks
**Platform**		GPL570		
**Number of control samples**		3	4	9
**Number of test samples**		23	4	9

* Pre-treated with TNF-α (10 ng/mL) for 48 h; ** TGF-β subtype (1/2/3) unspecified.

**Table 2:** Prognostic gene biomarkers in NSCLC, ranked according to their z-scores.

Gene (DEG)	Gene/Protein Function *	Module	FDR **	Z-Score
***KLHL25***	Substrate-specific adapter of a BCR E3 ubiquitin ligase complex, required for translational homeostasis	1	6.58 × 10^−3^	4.024
***FOXA2***	Transcription factor, involved in embryonic development and regulation of gene expression in differentiated tissues; inhibitor of EMT (Tang et al., 2011)	2	1.69 × 10^−4^	−5.316
***KCTD6***	Substrate-specific adapter of a BCR E3 ubiquitin ligase complex; downregulates HDAC1	1	2.23 × 10^−3^	−4.557
***KBTBD7***	Transcriptional activator, regulates the ubiquitination of a regulator of RAC1	1	2.30 × 10^−3^	−4.541
***DHTKD1***	Component of a mitochondrial 2-oxoglutarate-dehydrogenase-complex-like protein, catalyzes the overall conversion of 2-oxoglutarate to succinyl-CoA and CO_2_	2	5.60 × 10^−3^	−4.117
***LMO7***	Involved in protein ubiquitination and post-translational protein modification, regulates cell adhesion and signaling	1	1.19 × 10^−2^	−3.758
***PDHB***	Component of the pyruvate dehydrogenase complex, catalyzes the overall conversion of pyruvate to acetyl-CoA and CO_2_, linking glycolytic pathway to TCA cycle	3	1.72 × 10^−2^	−3.589
***SPSB2***	Substrate recognition component of an ECS E3 ubiquitin ligase complex, responsible for proteasomal degradation of proteins	1	2.07 × 10^−2^	−3.495
***CDH1***	E-cadherin, calcium-dependent intercellular adhesion molecule; the loss of its function is associated with carcinoma progression	2	3.20 × 10^−2^	−3.267
***RNF19A***	E3 ubiquitin ligase, specifically ubiquitinates pathogenic superoxide dismutase 1 (SOD1) variants	1	4.77 × 10^−2^	−3.075

* Sources: UniProt database https://www.uniprot.org/ (accessed on 11 May 2021) and NCBI Gene database https://www.ncbi.nlm.nih.gov/gene/ (accessed on 11 May 2021); ** *p* < 0.05 corrected by BH FDR.

## Data Availability

All relevant data generated during this study are included in this published article.

## Supplementary Materials

The following are available online at https://www.mdpi.com/article/10.3390/biology10111200/s1, Figure S1: Significant DEGs of the combined microarray datasets of TGF-β-induced EMT in NSCLC cell lines, Figure S2: Protein-protein interaction network of DEGs in EMT of NSCLC cells, Figure S3: Prognostic value of genes associated with EMT in NSCLC–Survival until first progression (FP), Figure S4: Prognostic value of genes associated with EMT in NSCLC–Post (first) progression survival (PPS), Table S1: List of the 20 most highly ranked upregulated genes in EMT of NSCLC cells with their z-scores, listed in order of their FDR values, Table S2: List of the 20 most highly ranked downregulated genes in EMT of NSCLC cells with their z-scores, listed in order of their FDR values, Table S3: Genes with their z-scores from the PPI network-based clustered modules in EMT of NSCLC cells, listed in order of their FDR values, Table S4: List of datasets sourced for patient survival analysis.
