# Supplementary material for: Identification of Prognostic Gene Biomarkers in Non-Small Cell Lung Cancer Progression by Integrated Bioinformatics Analysis
Source: Biology (Basel). 2021 Nov 18;10(11):1200. doi: 10.3390/biology10111200 (PMC8615219; doi:10.3390/biology10111200)
Supplement: Supplementary file 1 [file biology-10-01200-s001.zip › biology-1446674-new-supplementary material v3.pdf]

SUPPLEMENTARY MATERIAL

Figure S1: Significant DEGs of the combined microarray datasets of TGF-β-induced EMT in NSCLC cell lines

Figure S2: Protein-protein interaction network of DEGs in EMT of NSCLC cells

Figure S3: Prognostic value of genes associated with EMT in NSCLC – Survival until first progression (FP)

Figure S4: Prognostic value of genes associated with EMT in NSCLC – Post (first) progression survival (PPS)

Table S1: List of the 20 most highly ranked upregulated genes in EMT of NSCLC cells with their z-scores, listed in order of their FDR values

Table S2: List of the 20 most highly ranked downregulated genes in EMT of NSCLC cells with their z-scores, listed in order of their FDR values

Table S3: Genes with their z-scores from the PPI network-based clustered modules in EMT of NSCLC cells, listed in order of their FDR values

Table S4: List of datasets sourced for patient survival analysis

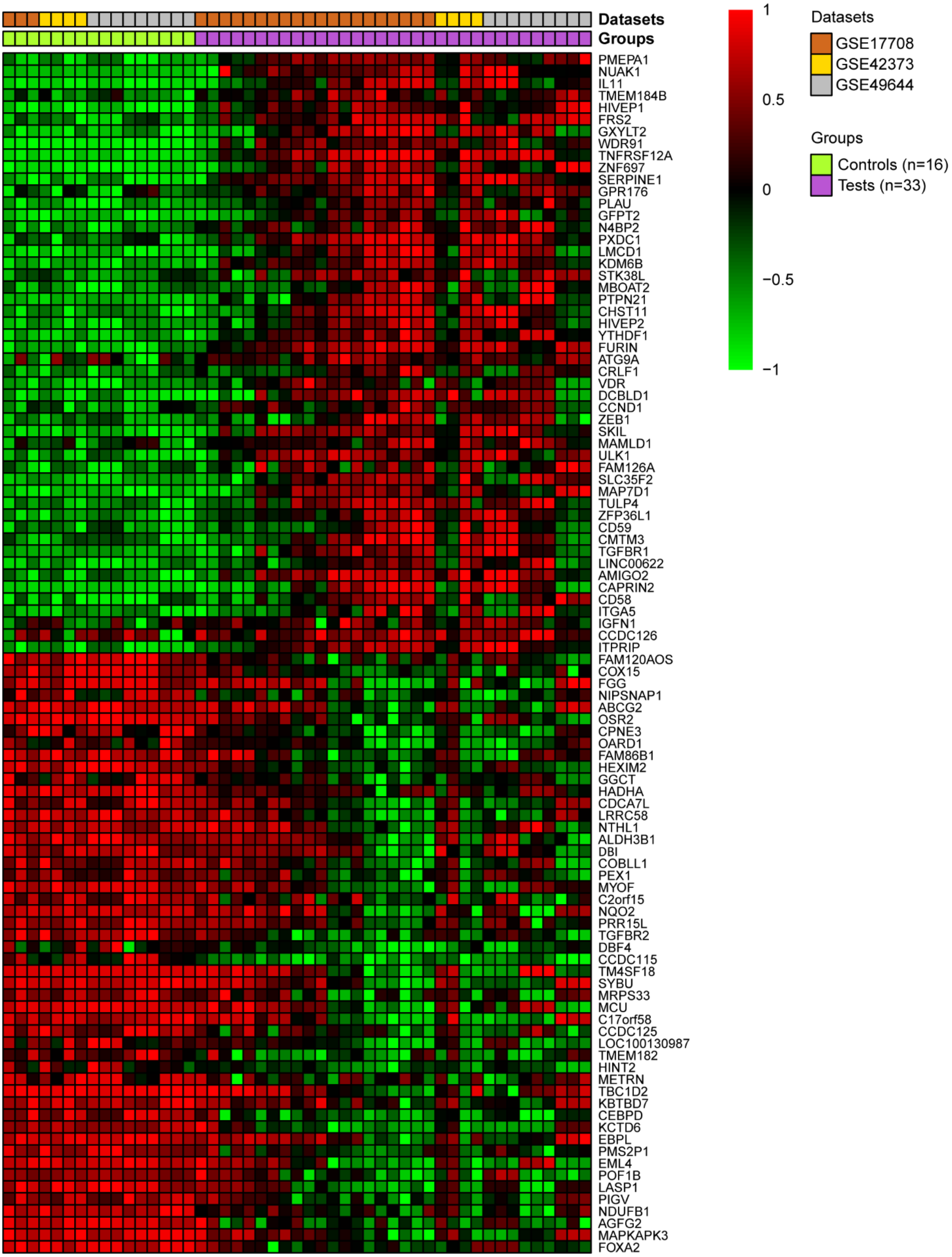

Figure S1. Significant DEGs of the combined microarray datasets of TGF-β-induced EMT in NSCLC cell lines. Heatmap of the top 100 DEGs from the combined microarray datasets listed according to their ranking based on their z-scores.

Table S1. List of the 20 most highly ranked upregulated genes in EMT of NSCLC cells with their z-scores, listed in order of their FDR values.

Gene symbol	Gene name	FDR*	Z-score
PMEPA1	prostate transmembrane protein, androgen induced 1	6.20×10^{-5}	5.888
NUAK1	NUAK family kinase 1	1.08×10^{-4}	5.679
FRS2	fibroblast growth factor receptor substrate 2	1.69×10^{-4}	5.332
GXYLT2	glucoside xylosyltransferase 2	1.69×10^{-4}	5.323
HIVEP1	human immunodeficiency virus type I enhancer binding protein 1	1.69×10^{-4}	5.372
IL11	interleukin 11	1.69×10^{-4}	5.402
WDR91	WD repeat domain 91	1.69×10^{-4}	5.320
ZNF697	zinc finger protein 697	3.69×10^{-4}	5.153
GPR176	G protein-coupled receptor 176	4.89×10^{-4}	5.041
GFPT2	glutamine-fructose-6-phosphate transaminase 2	5.99×10^{-4}	4.961
N4BP2	NEDD4 binding protein 2	5.99×10^{-4}	4.956
PLAU	plasminogen activator, urokinase	5.99×10^{-4}	4.966
PXDC1	PX domain containing 1	7.12×10^{-4}	4.913
LMCD1	LIM and cysteine rich domains 1	1.04×10^{-3}	4.810
KDM6B	lysine demethylase 6B	1.07×10^{-3}	4.795
STK38L	serine/threonine kinase 38 like	1.10×10^{-3}	4.774
PTPN21	protein tyrosine phosphatase, non-receptor type 21	1.23×10^{-3}	4.736
HIVEP2	human immunodeficiency virus type I enhancer binding protein 2	1.60×10^{-3}	4.669
YTHDF1	YTH N6-methyladenosine RNA binding protein 1	1.82×10^{-3}	4.629
ATG9A	autophagy related 9A	2.29×10^{-3}	4.539

* $p < 0.05$ corrected by BH FDR

Table S2. List of the 20 most highly ranked downregulated genes in EMT of NSCLC cells with their z-scores, listed in order of their FDR values.

Gene symbol	Gene name	FDR*	Z-score
FOXA2	forkhead box A2	1.69×10^{-4}	-5.316
AGFG2	ArfGAP with FG repeats 2	4.37×10^{-4}	-5.076
MAPKAPK3	mitogen-activated protein kinase-activated protein kinase 3	4.37×10^{-4}	-5.079
NDUFB1	NADH:ubiquinone oxidoreductase subunit B1	5.54×10^{-4}	-5.005
PIGV	phosphatidylinositol glycan anchor biosynthesis class V	7.41×10^{-4}	-4.895
LASP1	LIM and SH3 protein 1	7.58×10^{-4}	-4.882
EML4	echinoderm microtubule associated protein like 4	1.78×10^{-3}	-4.640
EBPL	emopamil binding protein like	2.00×10^{-3}	-4.596
PMS2P1	PMS1 homolog 2, mismatch repair system component pseudogene 1	2.00×10^{-3}	-4.600
CEBPD	CCAAT enhancer binding protein delta	2.23×10^{-3}	-4.556
KCTD6	potassium channel tetramerization domain containing 6	2.23×10^{-3}	-4.557
KBTBD7	kelch repeat and BTB domain containing 7	2.29×10^{-3}	-4.541
TBC1D2	TBC1 domain family member 2	2.55×10^{-3}	-4.511
METRN	meteorin, glial cell differentiation regulator	2.59×10^{-3}	-4.498
HINT2	histidine triad nucleotide binding protein 2	2.75×10^{-3}	-4.470
TMEM182	transmembrane protein 182	2.98×10^{-3}	-4.443
LOC100130987	uncharacterized LOC100130987	3.11×10^{-3}	-4.430
CCDC125	coiled-coil domain containing 125	3.22×10^{-3}	-4.418
C17orf58	chromosome 17 open reading frame 58	3.38×10^{-3}	-4.403
MCU	mitochondrial calcium uniporter	3.53×10^{-3}	-4.381

* $p < 0.05$ corrected by BH FDR

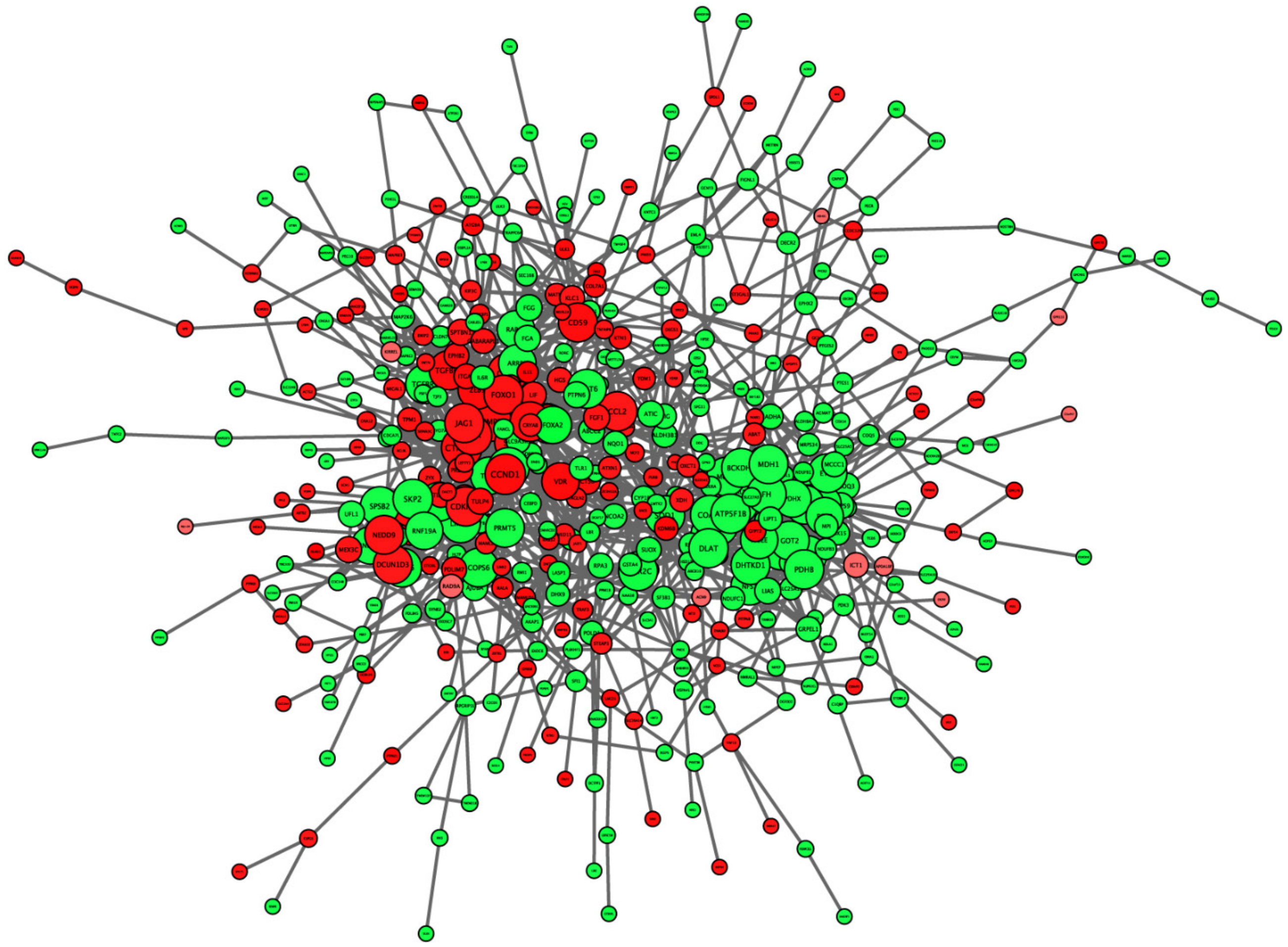

Figure S2. Protein-protein interaction network of DEGs in EMT of NSCLC cells. Network construction of proteins encoded by the total DEGs was carried out. All nodes with a combined interaction score > 0.4 are displayed, whereas isolated nodes are ignored. Larger nodes have a central regulatory role with a centrality degree ≥11.0. Red indicates upregulated and green shows downregulated node genes.

Table S3. Genes with their z-scores from the PPI network-based clustered modules in EMT of NSCLC cells, listed in order of their FDR values.

Gene symbol	Gene name	FDR*	Z-score
**Module 1**			
KCTD6	potassium channel tetramerization domain containing 6	2.23 x 10^{-3}	-4.557
KBTBD7	kelch repeat and BTB domain containing 7	2.29 x 10^{-3}	-4.541
TULP4	tubby like protein 4	4.88 x 10^{-3}	4.255
KLHL25	kelch like family member 25	6.58 x 10^{-3}	4.024
FBXO32	F-box protein 32	8.94 x 10^{-3}	3.911
COPS6	COP9 signalosome subunit 6	9.32 x 10^{-3}	-3.875
LMO7	LIM domain 7	1.19 x 10^{-2}	-3.758
SKP2	S-phase kinase associated protein 2	1.53 x 10-^{2}	-3.658
SPSB2	splA/ryanodine receptor domain and SOCS box containing 2	2.07 x 10^{-2}	-3.495
MEX3C	mex-3 RNA binding family member C	2.73 x 10^{-2}	3.347
RNF115	ring finger protein 115	2.96 x 10^{-2}	-3.306
UFL1	UFM1 specific ligase 1	3.78 x 10^{-2}	-3.187
RNF19A	ring finger protein 19A, RBR E3 ubiquitin protein ligase	4.77 x 10^{-2}	-3.075
DCUN1D3	defective in cullin neddylation 1 domain containing 3	4.81 x 10^{-2}	3.071
**Module 2**			
FOXA2	forkhead box A2	1.69 x 10^{-4}	-5.316
PLAU	plasminogen activator, urokinase	5.99 x 10^{-4}	4.966
CCND1	cyclin D1	2.82 x 10^{-3}	4.460
TGFBR1	transforming growth factor beta receptor 1	5.16 x 10^{-3}	4.205
FGG	fibrinogen gamma chain	5.26 x 10^{-3}	-4.162
MMP2	matrix metallopeptidase 2	5.60 x 10^{-3}	4.108
DHTKD1	dehydrogenase E1 and transketolase domain containing 1	5.60 x 10^{-3}	-4.117
CTNNB1	catenin beta 1	5.69 x 10^{-3}	4.098
KTN1	kinectin 1	6.45 x 10^{-3}	4.040
BMP2	bone morphogenetic protein 2	1.11 x 10^{-2}	3.790
FGA	fibrinogen alpha chain	1.24 x 10^{-2}	-3.736
TIMP3	TIMP metallopeptidase inhibitor 3	2.06 x 10^{-2}	3.500
GSTA4	glutathione S-transferase alpha 4	2.34 x 10^{-2}	-3.422
CDH2	cadherin 2	2.45 x 10^{-2}	3.395
SOD1	superoxide dismutase 1	2.45 x 10^{-2}	-3.392
MATN3	matrilin 3	2.96 x 10^{-2}	3.308
GOLM1	Golgi membrane protein 1	2.96 x 10^{-2}	-3.308
PDHX	pyruvate dehydrogenase complex component X	3.20 x 10^{-2}	-3.269
CDH1	cadherin 1	3.20 x 10^{-2}	-3.267
JAG1	jagged 1	3.57 x 10^{-2}	3.214
CDKN1A	cyclin dependent kinase inhibitor 1A	3.78 x 10^{-2}	3.187
NQO1	NAD(P)H quinone dehydrogenase 1	3.91 x 10^{-2}	-3.170
GPI	glucose-6-phosphate isomerase	3.99 x 10^{-2}	-3.160
CCL2	C-C motif chemokine ligand 2	4.01 x 10^{-2}	3.158
GCLC	glutamate-cysteine ligase catalytic subunit	4.04 x 10^{-2}	-3.154
MDH1	malate dehydrogenase 1	4.63 x 10^{-2}	-3.087
**Module 3**			

TXNRD1	thioredoxin reductase 1	1.63×10^{-2}	-3.617
PDHB	pyruvate dehydrogenase E1 beta subunit	1.72×10^{-2}	-3.589
DLAT	dihydrolipoamide S-acetyltransferase	2.20×10^{-2}	-3.461
FH	fumarate hydratase	2.89×10^{-2}	-3.322
GSR	glutathione-disulfide reductase	3.84×10^{-2}	-3.177
**Module 4**			
MRPS33	mitochondrial ribosomal protein S33	3.72×10^{-3}	-4.362
MRPL24	mitochondrial ribosomal protein L24	7.27×10^{-3}	-3.980
MRPS34	mitochondrial ribosomal protein S34	1.72×10^{-2}	-3.591
MRPL58	mitochondrial ribosomal protein L58	3.61×10^{-2}	-3.209
MRPS9	mitochondrial ribosomal protein S9	3.92×10^{-2}	-3.168

* $p < 0.05$ corrected by BH FDR

Table S4. List of datasets sourced for patient survival analysis.

OS	FP	PPS
caArray	caArray	caArray
GSE14814	GSE29013	GSE29013
GSE19188	GSE31210	GSE31210
GSE29013	GSE31908	GSE31908
GSE30219	GSE50081	GSE50081
GSE31210	GSE8894	
GSE3141		
GSE31908		
GSE37745		
GSE4573		
GSE50081		
TCGA		

OS: overall survival; FP: survival until first progression;
PPS: post (first) progression survival

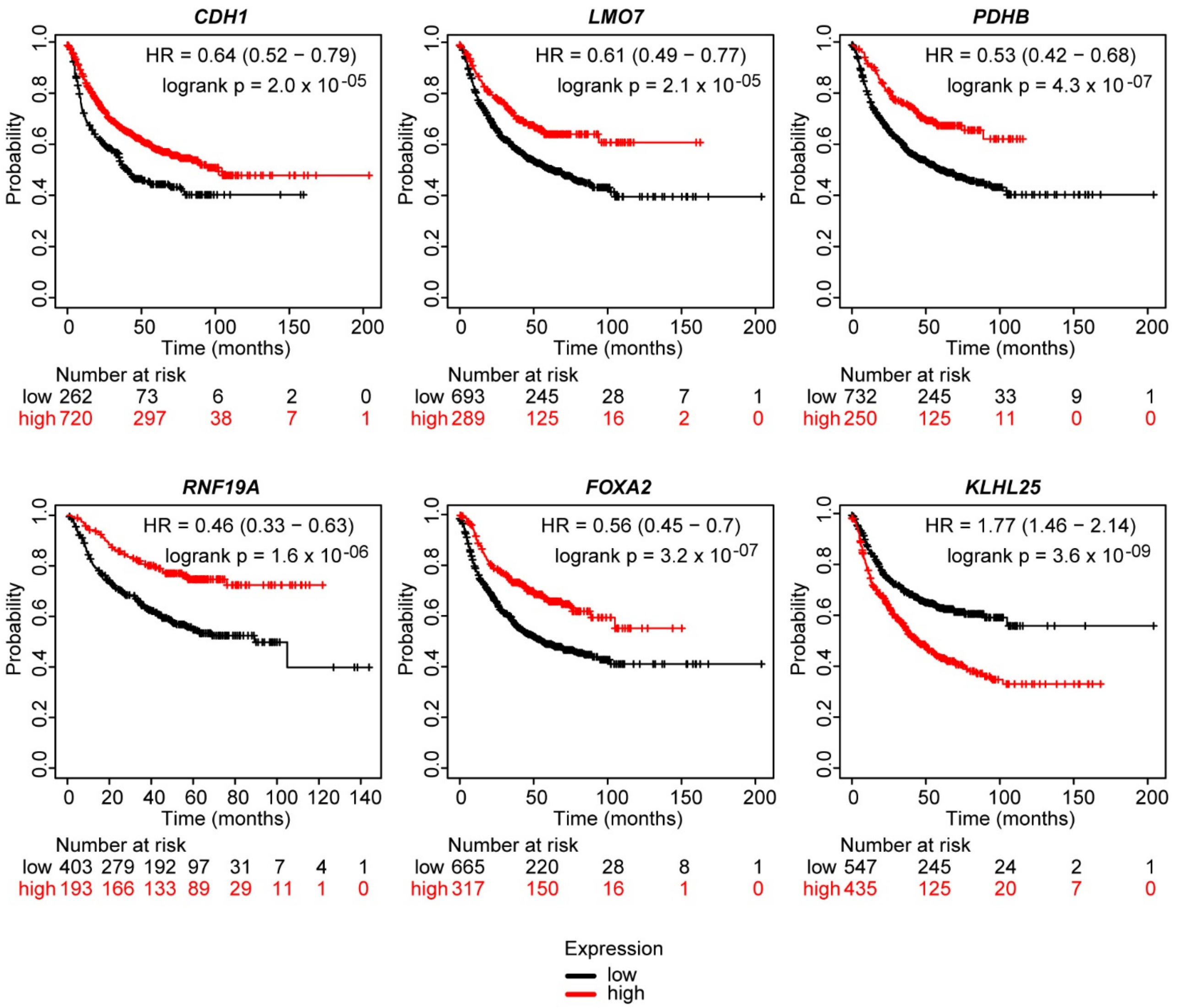

Figure S3. Prognostic value of genes associated with EMT in NSCLC – Survival until first progression (FP). Kaplan–Meier survival curves showing the correlation between marker gene expression and survival until FP in patients with NSCLC (n=596). Significance thresholds for potential gene biomarkers include a log-rank test $p < 0.001$ and a corrected $p < 0.05$ by BH FDR for the patient cut-off selection method. Low expression levels of *CDH1*, *LMO7*, *PDHB*, *RNF19A* and *FOXA2* whereas high expression level of *KLHL25* were associated with reduced survival until FP. HR: hazard ratio.

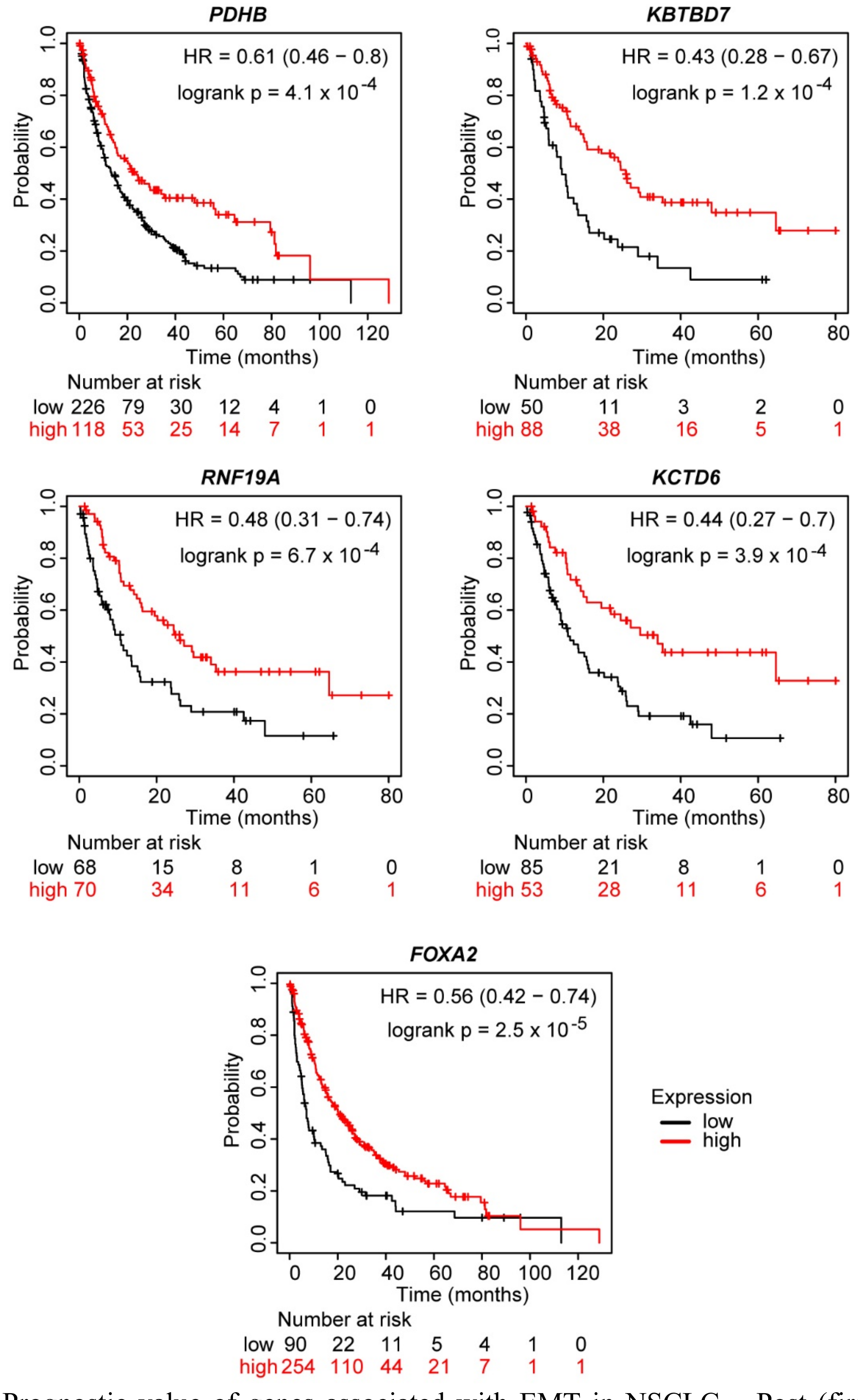

Figure S4. Prognostic value of genes associated with EMT in NSCLC – Post (first) progression survival (PPS). Kaplan–Meier survival curves showing the correlation between marker gene expression and PPS in patients with NSCLC (n=138). Significance thresholds for potential gene biomarkers include a log-rank test $p < 0.001$ and a corrected $p < 0.05$ by BH FDR for the patient cut-off selection method. Low expression levels of *PDHB*, *KBTBD7*, *RNF19A*, *KCTD6* and *FOXA2* were associated with reduced PPS. HR: hazard ratio.
